# Impact of varying social experiences during life history on behaviour, gene expression, and vasopressin receptor gene methylation in mice

**DOI:** 10.1038/s41598-017-09292-0

**Published:** 2017-08-18

**Authors:** Carina Bodden, Daniel van den Hove, Klaus-Peter Lesch, Norbert Sachser

**Affiliations:** 1 Department of Behavioural Biology, University of Münster, Münster, Germany; 2 Otto Creutzfeldt Center for Cognitive and Behavioral Neuroscience, University of Münster, Münster, Germany; 3 Division of Molecular Psychiatry, Center of Mental Health, Laboratory of Translational Neuroscience, University of Würzburg, Würzburg, Germany; 4 Department of Translational Neuroscience, School for Mental Health and Neuroscience, Maastricht University, Maastricht, The Netherlands; 5 Laboratory of Psychiatric Neurobiology, Institute of Molecular Medicine, I.M. Sechenov First Moscow State Medical University, Moscow, Russia

## Abstract

Both negative and positive social experiences during sensitive life phases profoundly shape brain and behaviour. Current research is therefore increasingly focusing on mechanisms mediating the interaction between varying life experiences and the epigenome. Here, male mice grew up under either adverse or beneficial conditions until adulthood, when they were subdivided into groups exposed to situations that either matched or mismatched previous conditions. It was investigated whether the resulting four life histories were associated with changes in anxiety-like behaviour, gene expression of selected genes involved in anxiety and stress circuits, and arginine vasopressin receptor 1a (*Avpr1a*) gene methylation. Varying experiences during life significantly modulated (1) anxiety-like behaviour; (2) hippocampal gene expression of *Avpr1a*, serotonin receptor 1a (*Htr1a*), monoamine oxidase A (*Maoa*), myelin basic protein (*Mbp*), glucocorticoid receptor (*Nr3c1*), growth hormone (*Gh*); and (3) hippocampal DNA methylation within the *Avpr1a* gene. Notably, mice experiencing early beneficial and later adverse conditions showed a most pronounced downregulation of *Avpr1a* expression, accompanied by low anxiety-like behaviour. This decrease in *Avpr1a* expression may have been, in part, a consequence of increased methylation in the *Avpr1a* gene. In summary, this study highlights the impact of interactive social experiences throughout life on the hippocampal epigenotype and associated behaviour.

## Introduction

An individual’s phenotype is the result of a complex interplay between its genotype and the environment. Experiences during early phases of life have long been known to profoundly modulate behavioural and transcriptomic profiles^1^. Over the past years, a growing body of evidence also revealed that experiences during later phases of life can significantly impact upon an individual’s epigenotype, thereby mediating behavioural variations^2^. More recently, the focus has moved from investigating the influence of experiences during single phases of life to the study of whole life histories both in rodents and humans^3–5^.

In our previous work in mice, we first demonstrated that adverse social experiences during different phases of life modulate not only anxiety-like behaviour and exploratory locomotion^6–9^, but also neuronal morphology^10^, fear conditioning and extinction as well as corresponding neurophysiologic response patterns^11^. In subsequent studies, we combined several established paradigms to create adverse and beneficial experiences in a whole-life-history approach^3, 5^. As a main result, limited adversity during adulthood was associated with decreased anxiety-like and increased exploratory behaviour, however, only in combination with beneficial experiences during early phases of life. In line with findings from human and non-human primate research, it was argued that some lifetime adversity may be beneficial^3, 12, 13^.

Although numerous studies have already reported long-term effects of early social experiences, the mechanisms mediating these effects are not well understood. In this context, it is also not clear how experiences during later phases of life exert effects on brain function and behaviour^14, 15^. Recent evidence indicates that environmental influences can induce changes in the epigenome, which is comprised of DNA methylation and chromatin structure, thereby inducing transcriptional alterations^16–19^. Although reversible in nature, these epigenetic changes often persist beyond infancy even into adulthood, representing a molecular pathway through which long-term-programming effects are achieved^16^. Yet again, epigenetic regulation appears to be dynamic, where effects of early life experiences may be changed again by environmental variation later in life. This makes the study of the interplay between genes and experiences across the lifespan extremely important for environmental and pharmacologic intervention^20, 21^.

Over the past years, an increasing number of rodent studies demonstrated that epigenetic mechanisms can modulate various signalling pathways that are involved in the regulation of emotional behaviours and hypothalamic-pituitary-adrenal (HPA) responses to stress^1, 17, 21–23^. One of these pathways is serotonergic neurotransmission, which plays a key modulatory role in central nervous system processes, underlying for example anxiety, fear, and aggression^24^. Among the elements of the serotonin system identified as crucial regulators of emotional behaviours are the serotonin receptors 1a and 2a (*Htr1a*, *Htr2a*)^25, 26^ as well as monoamine oxidase A (*Maoa*)^27^. Elements known for their modulatory function of the HPA axis include the glucocorticoid receptor (GR; *Nr3c1*)^17, 28^, corticotropin releasing hormone receptor 1 (*Crhr1*)^29, 30^, and brain-derived neurotrophic factor (*Bdnf*)^31, 32^. Furthermore, growth hormone (*Gh*) and myelin basic protein (*Mbp*) appeared as candidate genes in previous studies^33–35^. Both play important roles in the brain as they are involved in neural processes such as myelination. Additionally, central pathways for vasopressin and oxytocin have been implicated in the neurobiologic mechanisms underlying anxiety and social behaviours. Hence, arginine vasopressin receptor 1a (*Avpr1a*) and oxytocin receptor (*Oxtr*) have garnered much attention since there is also increasing evidence for effects of social interactions, in particular social defeat, on their expression levels^36–38^. Since social experiences often involve testosterone release, which acts through androgenic pathways, the androgen receptor (*Ar*) has raised interest. Notably, *Ar* expression levels appear to be profoundly influenced by sexual behaviour as well as winning fights^39, 40^. Moreover, the social status (‘dominant’ vs ‘submissive’) was found to be reflected in gene expression levels of neuropeptide Y (*Npy*) and *Bdnf* ^41^.

So far, it is well-known that experiences during specific phases of life profoundly influence an individual’s epigenome, however, the impact of whole life histories is still unknown. Based on our previous work^3, 5^, we hypothesized that variations in life histories bring about not only variations in the behavioural but also in the epigenetic profile. Consequently, we investigated the influence of different combinations of adverse and beneficial experiences throughout life on gene expression and DNA methylation as well as anxiety-like behaviour and exploratory locomotion in male C57BL/6 J mice.

## Methods

### Animals and housing conditions

In the present study, we used male C57BL/6 J mice (n = 63), which were bred in the Department of Behavioural Biology at the University of Münster, Germany. All animals were housed in transparent standard Makrolon cages type III (38 × 22 × 15 cm) with sawdust as bedding material and a paper towel as nesting material. Food pellets and tap water were provided *ad libitum*. Housing rooms were maintained at a 12 h light/dark cycle with lights on at 8:00 a.m., a temperature of about 22 °C, and a relative air humidity of about 50%.

### Ethics statement

All procedures complied with the regulations covering animal experimentation within the EU (European Communities Council DIRECTIVE 2010/63/EU). They were conducted in accordance with the institutional and national guidelines for the care and use of laboratory animals and approved by the local government authorities (Landesamt für Natur, Umwelt und Verbraucherschutz Nordrhein-Westfalen, LANUV NRW, reference number: 84–02.05.20.12.212).

### Experimental design

**Figure 1:**
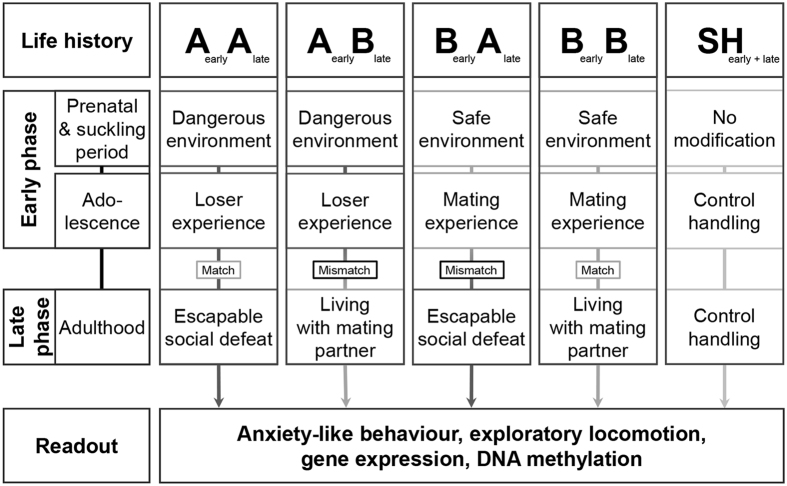
Experimental design. Mice were assigned to one of the four life histories or the sham-handled group. They were provided with either matching or mismatching adverse (‘A’) and/or beneficial (‘B’) conditions from the prenatal phase to adulthood, or a sham-handling (‘SH’) procedure at corresponding times. Anxiety-like and exploratory behaviour were assessed. Afterwards, brains were dissected and subjected to gene expression and methylation analyses (Figure modified after^3, 5^).

Four different life histories (group size AA: 13, AB: 12, BA: 13, BB: 12) were experimentally induced according to the paradigm published earlier^3^. In line with a follow-up study^5^, an additional sham-handled group was included to control for handling effects (group size SH: 13). Mice were randomly assigned to one of the five experimental groups. Briefly, life histories were divided into an early and a late phase (see Fig. 1). The early phase comprised the prenatal and suckling period as well as adolescence, while the subsequent phase of early adulthood was referred to as the late phase. During both the early and late phase, mice were exposed to either adverse (‘A’) or beneficial (‘B’) experiences, or a sham-handling procedure (‘SH’). Thereby, animals underwent either consistent (AA, BB) or changing (AB, BA) social experiences from the prenatal stage until adulthood.

Adverse experiences (A) during the early phase were created by two different consecutive treatments. First, during the prenatal and suckling period (AA and AB), an adverse environment was created by exposing pregnant and lactating female C57BL/6 J mice to soiled bedding from unfamiliar males. These olfactory cues signal the danger of infant killing and thus, simulate a dangerous environment^6, 9^. Second, after being separated from the mother on postnatal day (PND) 22, the male offspring were housed in same-sex groups of two to five animals until PND 35 ± 2. From then on, mice were housed individually. During adolescence, adverse experiences (AA and AB) were generated by confronting the male subjects with an adult male mouse from the aggressive NMRI strain^42^ in order to provide loser experiences^7^ (5 times, 10 min duration, PND 37 ± 2–61 ± 2).

Beneficial conditions (B) during the early phase (BA and BB) were also created by two consecutive treatments. First, pregnant and lactating female C57BL/6 J mice were exposed to neutral bedding in order to simulate a safe environmental setting. Second, with the onset of adolescence, beneficial experiences for the male offspring (BA and BB) were provided by repeated encounters with a female mouse in oestrus, allowing for mating experiences, which has been shown to reduce anxiety-like behaviour^43^ (5 times, 10 min duration, PND 37 ± 2–61 ± 2).

During adulthood, the male test subjects were transferred to a custom-made cage system, where they experienced either an escapable adverse (AA and BA) or a beneficial situation (AB and BB; PND 70 ± 2). The cage system provided the opportunity to escape from potential agonistic encounters in the main cage to another ‘safe’ cage via a water basin (filled to a height of approximately 3 cm)^8^. After an acclimatization phase of 24 hours, in which the experimental male was allowed to freely explore the cage system alone, either an aggressive male mouse from the NMRI strain (applied to AA and BA mice) or a female C57BL/6 J mouse in oestrus (applied to AB and BB mice) was additionally placed into the main cage (PND 71 ± 2; for details see ref. 3). On 6 out of the following 8 days, (PND 72 ± 2–79 ± 2), the experimental animal was either placed back into the main cage where the aggressive NMRI male resided (AA and BA) or shortly handled and returned to the female (AB and BB). As a result, AA and BA mice repeatedly experienced escapable social defeat, while AB and BB animals were constantly living with a mating partner.

SH animals were not provided with any environmental modifications in their life history (neutral environment). Instead of social encounters during adolescence and adulthood, these animals were sham-handled by the experimenter at the corresponding times.

### Behavioural tests

Behavioural testing took place between PND 75 ± 2 and 77 ± 2 days. In total, 63 males (sample size: AA: 13, AB: 12, BA: 13, BB: 12, SH: 13) were investigated for their anxiety-like and exploratory behaviour in the Elevated plus-maze test (EPM)^44^, the Dark-light test (DL)^45^, and the Open-field test (OF)^46^. The order of tests was the same for each animal with one test per day. All behavioural tests lasted 5 min and were performed during the light phase in a room different from the housing room. The test equipment was cleaned with 70% ethanol between subjects. The animal’s movements were recorded by a webcam (Logitech Webcam Pro 9000, Logitech Europe S.A., Lausanne, Switzerland) and analysed by the video tracking system ANY-maze (Version 4.75, Stoelting Co., Wood Dale, USA). Please note that the behavioural data of the present work are a subset of the data published earlier^5^. For more details on procedures, see Supplementary Methods.

#### Elevated plus-maze test

The parameters measured were the percentage of time spent on the open arms as well as the percentage of entries into the open arms to assess anxiety-like behaviour, and the sum of entries into open and closed arms as an indicator of exploratory locomotion.

#### Dark-light test

The parameters analysed were the latency to enter the light compartment and the percentage of time spent in the light compartment as indicators of anxiety-like behaviour, and the number of entries into the light compartment to assess exploratory locomotion.

#### Open-field test

The parameters analysed were the time spent in the centre of the arena (defined as the area of the OF being located at least 20 cm distant from the walls) to measure anxiety-like behaviour and the distance travelled for assessing exploratory locomotion.

### Gene expression and DNA methylation analysis

Concerning gene expression analysis, we concentrated on 13 candidate genes that have been associated with HPA axis (re)activity, anxiety-like and social behaviours, brain myelination, and neuronal and synaptic plasticity. In total, brains of 61 mice were used (n = 8–13/group).

#### Tissue preparation

On PND 79 ± 2, two hours after the last handling in the cage system, mice were anesthetized using 2.5% isoflurane in oxygen and decapitated. Brains were immediately removed and frozen on dry ice. Afterwards, the left and right part of the hippocampus and the amygdala, respectively, were dissected on a cooling plate and subsequently crushed on dry ice to homogenize the tissue. Half of the crushed tissue was used for RNA extraction, and the other half was used for DNA extraction. Due to technical reasons, the sample sizes partly differed (gene expression analysis: AA: 13, AB: 12, BA: 11–12, BB: 11, SH: 13; DNA methylation analysis: AA: 13, AB: 8–12, BA: 11, BB: 11, SH: 12–13).

#### Gene expression analysis

Gene expression was analysed via quantitative real-time PCR (qRT-PCR) employing the Bio-Rad CFX384 Real-Time PCR Detection System (in technical triplicates). Mean efficiencies were calculated by LinReg^47^. Reference genes for normalization were tested for stability using geNorm^48^. Guanosine diphosphate *(Gdp)* dissociation inhibitor 2 *(Gdi2)*, TATA-binding protein *(Tbp)*, and Smad nuclear interacting protein 1 *(Snip1)* were used for normalization. Reference gene stability was very high (geNorm M < 0.2) and expression levels were neither affected by life history nor by the early or late phase. Relative expression data were calculated by qbase+ (Biogazelle, Zwijnaarde, Belgium), taking into account the normalization factors obtained from geNorm and the mean efficiencies from LinReg. More details on procedures as well as primer sequences can be found as Supplementary Methods and Table S1.

#### DNA methylation analysis

**Figure 2:**
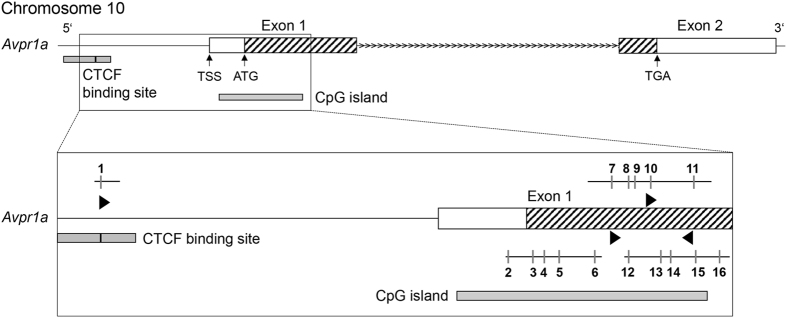
Map of the *Avpr1a* gene. Triangles indicate sequences analysed for DNA methylation. Vertical grey lines mark independent CpG sites (1–16). TSS = transcription start site; ATG = translation start codon; TGA = stop codon; CTCF = 11-zinc finger protein or CCCTC-binding factor (Figure created based on information from UCSC Genome Browser^69^, Ensembl^70^, and Qiagen).

DNA bisulphite treatment was performed using the EpiTect® Fast DNA Bisulfite Kit (Qiagen) according to the manufacturer’s instructions. The PyroMark CpG Assays (Mm_AVPR1a_01_PM [PM00220241], Mm_AVPR1a_02_PM [PM00220248], Mm_AVPR1a_04_PM [PM00220262]) were obtained from Qiagen. Further PCR and sequencing primers (Mm_AVPR1a_TF) were designed with the PyroMark Assay Design 2.0.1.15 Software (Qiagen; see Fig. 2 for gene map). Pyrosequencing was conducted using the PyroMark Q96 MD™ Pyrosequencer (Biotage, Uppsala, Sweden) and PyroMark Gold Q96 CDT Reagents Kit (Qiagen). Results were analysed with the PyroMark CpG software (Qiagen). For more details on procedures as well as sequences analysed, see Supplementary Methods and Table S2.

### Statistics

General Linear Models (GLM) were used to analyse data obtained from tests for anxiety-like behaviour and exploratory locomotion as well as gene expression and DNA methylation data. Residuals were graphically examined for homoscedasticity and outliers and the Lilliefors corrected Kolmogorov-Smirnov Test was applied. Though not all parameters met the assumptions of parametric analysis, GLM were used since ANOVA is not very sensitive to moderate deviations from normality, as indicated by simulation studies using a variety of non-normal distributions, which showed that the false positive rate is only minimally affected by this violation of the assumption^49–51^. Specifically, the following kinds of models were established and fitted to the different dependent variables:

**Model (A)** Life history analysis. Univariate ANOVA of several dependent variables (anxiety-like and exploratory behaviours, gene expression, DNA methylation) with fixed between-subject factor ‘life history’. This analysis served to detect main effects of whole life histories.

**Model (B)** Early vs late phase analysis (without group SH). Univariate ANOVA of several dependent variables (anxiety-like and exploratory behaviours, gene expression and DNA methylation) with fixed between-subject factors ‘early phase’ (adverse: AA and AB; beneficial: BA and BB), ‘late phase’ (adverse: AA and BA; beneficial: AB and BB), and the interaction of ‘early phase’ and ‘late phase’. This analysis served to disentangle specific effects of the early vs late phase. A significant interaction would be indicative of a mismatch effect.

All main effects and interaction terms were tested on local significance level alpha = 0.05, respectively. If there were significant main or interaction effects, post hoc pairwise comparisons of different levels were conducted using Bonferroni adjustment. Data are presented as bars with means and standard error (s.e.m).

All statistical analyses were conducted using the statistical software IBM SPSS Statistics (IBM Version 23, Release 2015). Graphs were created using the software SigmaPlot for Windows (Version 12.5, Build 12.5.0.38, Systat Software, Inc. 2011).

### Data availability

All data generated or analysed during this study are included in this published article (and its Supplementary Information files).

## Results

### Anxiety-like behaviour and exploratory locomotion

#### Effects of life history

**Figure 3:**
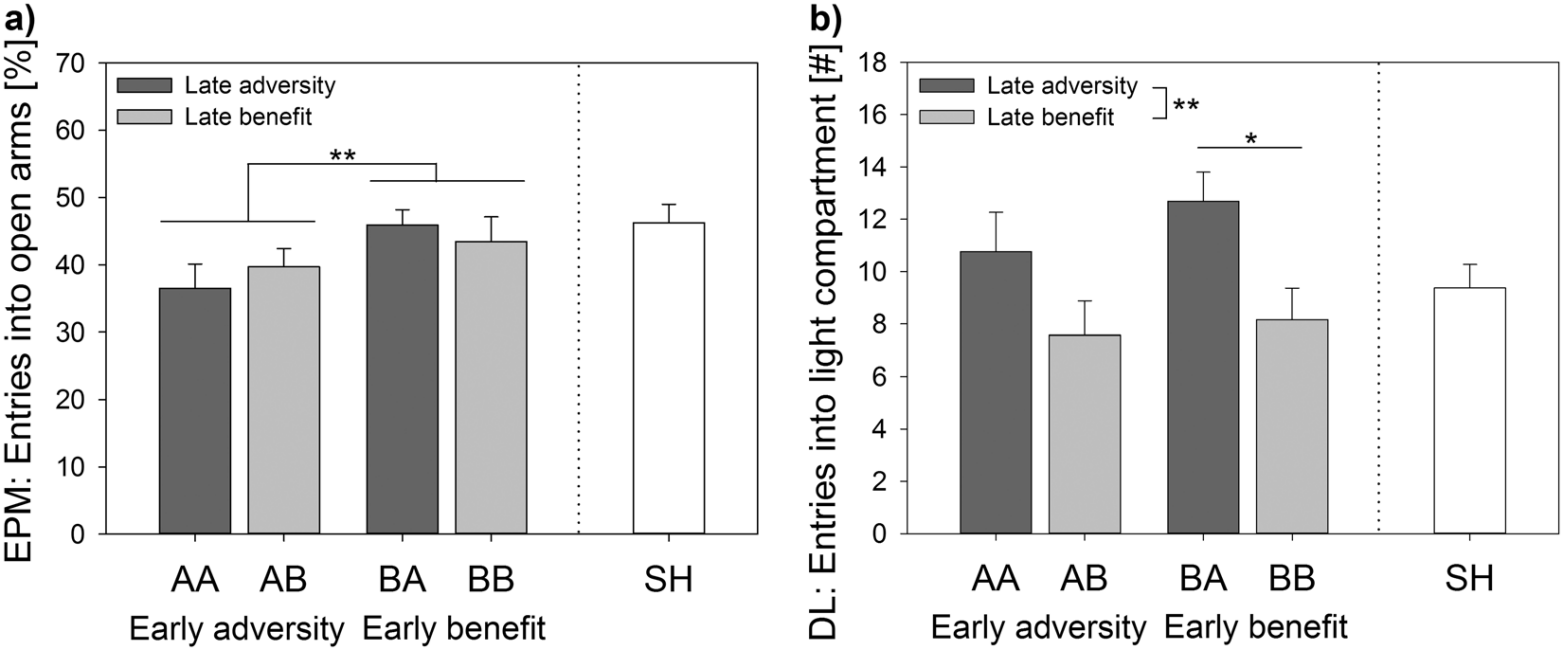
Anxiety-like behaviour and exploratory locomotion. (**a**) Percentage of entries into open arms of Elevated plus-maze (EPM) test and (**b**) Entries into light compartment of Dark-light (DL) test displayed by mice grown up in an early adverse (AA and AB) or beneficial (BA and BB) environment and provided with later matching (AA and BB) or mismatching (AB and BA) conditions in adulthood. Sham-handled mice (SH) served as control. There was a significant main effect of the early phase on the percentage of entries into the open arms (p = 0.042) as well as a significant main effect of life history (p = 0.031) and the late phase (p = 0.005) on the entries into the light compartment. Data are given as means + s.e.m. Statistics: ANOVA, Bonferroni *post-hoc* testing. *p ≤ 0.05; **p ≤ 0.01. n = 12–13.

A significant main effect of life history on anxiety-like behaviour was detected as revealed by the time in the centre of the OF (Model A, F_(4,58)_ = 2.632, p = 0.043; see Supplementary Table S3). Regarding exploratory locomotion, there was a significant main effect of life history as reflected by the sum of entries in the EPM (F_(4,58)_ = 2.783, p = 0.035), entries into the light compartment of the DL (F_(4,58)_ = 2.868, p = 0.031; Fig. 3b), and distance travelled in the OF (F_(4,58)_ = 3.293, p = 0.017). More specifically, AB mice entered the light compartment significantly less often than BA mice (p = 0.045) and travelled less in the OF than SH animals (p = 0.014; see Supplementary Table S4).

#### Effects of early vs late phase

The additional early vs late phase analysis revealed a significant main effect of the early phase on anxiety-like behaviour, as demonstrated by the percentage of entries into open arms in the EPM (Model B, F_(1,46)_ = 4.398, p = 0.042; Fig. 3a). More specifically, early adverse conditions increased anxiety-like behaviour. Furthermore, in particular the late phase was found to significantly influence exploratory locomotion, with late escapable adversity increasing the sum of entries into open and closed arms in the EPM (F_(1,46)_ = 10.313, p = 0.002), the number of entries into the light compartment of the DL (F_(1,46)_ = 8.912, p = 0.005; Fig. 3b), and the distance travelled in the OF (F_(1,46)_ = 5.409, p = 0.025).

### Gene expression

#### Effects of life history

**Figure 4:**
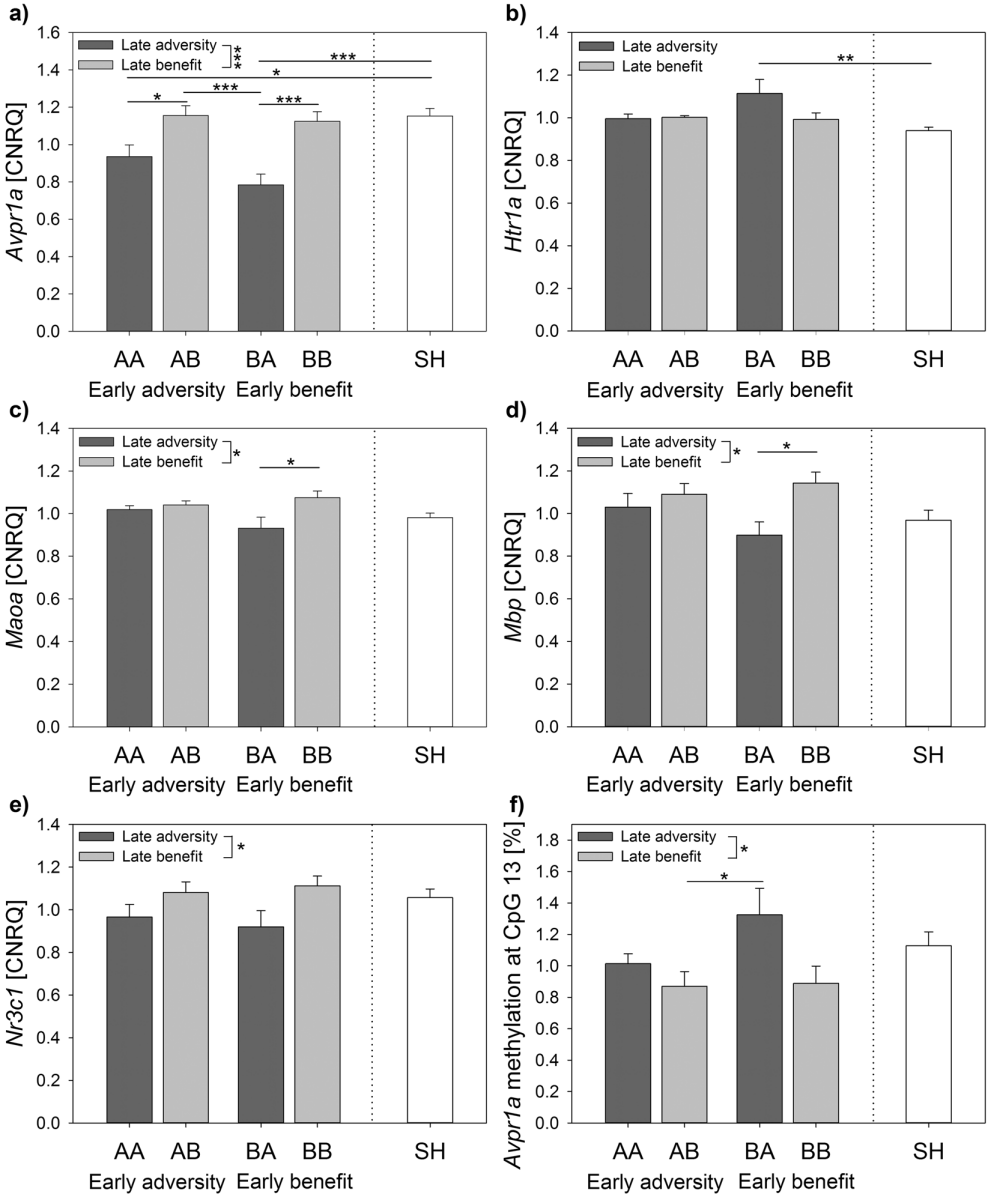
Gene expression and DNA methylation in the hippocampus. (**a–e**) Quantitative real-time polymerase chain reaction (qRT-PCR) analysis of (**a**) *Avpr1a*, (**b**) *Htr1a*, (**c**) *Maoa*, (**d**) *Mbp*, and (**e**) *Nrc31* mRNA as well as (**f**) pyrosequencing analysis of bisulphite-treated DNA at CpG site 13 within the *Avpr1a* gene of mice grown up in an early adverse (AA and AB) or beneficial (BA and BB) environment and provided with later matching (AA and BB) or mismatching (AB and BA) conditions in adulthood. Sham-handled mice (SH) served as control. There were significant main effects of life history (Gene expression: *Avpr1a*: p < 0.001; *Htr1a*: p = 0.012; *Maoa:* p = 0.020; *Mbp:* p = 0.033; DNA methylation: *Avpr1a* CpG site 13: p = 0.028) and of the late phase (Gene expression: *Avpr1a*: p < 0.001; *Maoa:* p = 0.014; *Mbp:* p = 0.013; *Nr3c1*: p = 0.013; DNA methylation: *Avpr1a* CpG site 13: p = 0.013). Data are given as means + s.e.m. Statistics: ANOVA, Bonferroni *post-hoc* testing. *p ≤ 0.05; **p ≤ 0.01; ***p ≤ 0.001. n = 11–13 (**a**–**e**), n = 8–13 (**f**). CNRQ = Calibrated Normalized Relative Quantities.

There were significant main effects of life history on hippocampal gene expression levels of *Avpr1a* (Model A, F_(4,56)_ = 9.501, p < 0.001; Fig. 4a), *Htr1a* (F_(4,56)_ = 3.533, p = 0.012; Fig. 4b), *Maoa* (F_(4,56)_ = 3.196, p = 0.020; Fig. 4c), and *Mbp* (F_(4,56)_ = 2.832, p = 0.033; Fig. 4d). Remarkably, expression patterns in BA mice stood out compared to mice of other life histories, as demonstrated by *post hoc* analyses. Differences between life histories were most pronounced regarding *Avpr1a* expression. BA mice had significantly lower *Avpr1a* expression levels in comparison to AB (p < 0.001), BB (p < 0.001), and SH mice (p < 0.001). Moreover, AA mice differed in their *Avpr1a* expression in terms of decreased levels compared to both AB (p = 0.047) and SH mice (p = 0.043). Concerning *Htr1a*, BA mice differed from SH mice in terms of higher expression levels (p = 0.006). *Maoa* and *Mbp* expression levels were found to be decreased in BA mice compared to BB animals (*Maoa:* p = 0.020; *Mbp:* p = 0.043).

Regarding gene expression of the analysed genes in the amygdala, no main effects of life history were found (see Supplementary Table S3).

#### Effects of early vs late phase

The additional early vs late phase analysis revealed significant main effects of the late phase on hippocampal gene expression of *Avpr1a* (Model B, F_(1,44)_ = 24.323, p < 0.001; Fig. 4a), *Maoa* (F_(1,44)_ = 6.514, p = 0.014; Fig. 4c), *Mbp* (F_(1,44)_ = 6.769, p = 0.013; Fig. 4d), and *Nr3c1* (F_(1,44)_ = 6.658, p = 0.013; Fig. 4e), with late escapable adversity being associated with lower expression levels when compared to late beneficial conditions. A significant early-by-late phase interaction was found regarding *Gh* expression (F_(1,43)_ = 5.394, p = 0.025). *Post-hoc* testing showed that late adversity was only associated with increased *Gh* expression, when following on beneficial but not when following on adverse early conditions (p = 0.010). Furthermore, early beneficial conditions only led to increased *Gh* expression, when followed by adverse late experiences but not when followed by beneficial late conditions (p = 0.019). The early vs late phase analysis did not reveal any significant main effects of the early phase on expression levels of the genes analysed.

Regarding gene expression in the amygdala, the early vs late phase analysis showed that the late phase significantly influenced *Npy* expression (F_(1,44)_ = 4.248, p = 0.045), with late adverse experiences predicting increased expression levels.

### DNA methylation

Since the most pronounced effects of life history were found concerning *Avpr1a* gene expression, we additionally investigated methylation within the *Avpr1a* gene at 16 CpG sites in the hippocampus (see Fig. 2).

#### Effects of life histories

A significant main effect of life history on methylation was detected for CpG site 13 (Model A, F_(4,54)_ = 2.957, p = 0.028), with increased methylation in BA mice compared to AB mice (p = 0.045; Fig. 4f).

#### Effects of early vs late phase

The early vs late phase analysis revealed significant main effects of the late phase on DNA methylation at three CpG sites (Model B, CpG site 6: F_(1,38)_ = 4.758, p = 0.035; CpG site 8: F_(1,42)_ = 4.052, p = 0.050; CpG site 13: F_(1,42)_ = 6.768, p = 0.013; Fig. 4f). While late adversity predicted increased methylation at CpG sites 6 and 13, methylation was decreased at CpG site 8 compared to late beneficial experiences. No significant main effect of the early phase was found, neither an early-by-late phase interaction.

## Discussion

On the behavioural level, we confirmed our earlier finding that life history profoundly modulates the behavioural profile^3, 5^. More precisely, we could validate that a beneficial early life followed by escapable adversity in later life (BA) is associated with relatively low levels of anxiety-like and relatively high levels of exploratory behaviour. The experimental design also allowed for testing the mismatch hypothesis, which predicts adverse consequences for the individual if the environmental experiences during early phases of life differ from those during later life stages^52^. However, the present results contradict the mismatch hypothesis since BA mice experienced mismatching conditions over the lifespan, but displayed diminished anxiety-like behaviour, which is in line with our previous findings. Strikingly, in addition to the behavioural effects of life history, we found significant alterations in gene expression levels. Again, mice of the BA group displayed a profoundly different expression pattern than mice of other life histories. Further analysis, performed to disentangle the effects of the early versus late phase, revealed that expression levels of several genes were significantly influenced by the late phase or, in one case, by an early-by-late phase interaction, whereas no effects of the early phase could be detected. Although it has been shown that early epigenetic marks can persist into adulthood, there is also evidence for plasticity, leaving the possibility that early effects were reversed by late effects^16, 20, 21^. In this study, differences in gene expression were only detected in the hippocampus. This brain region is fundamental in processing emotions, stress responses as well as spatial memory, and shows an impressive capacity for structural reorganization that is maintained even into adulthood^1, 22^ (for a review see ref. 53).

A key modulatory role in central nervous system processes, mediating anxiety and fear is provided by the serotonin system. In the hippocampus, HTR1A is the most abundant serotonin receptor and an important regulator of anxiety^25, 54, 55^. Here, we found a significant effect of life history on *Htr1a* expression, with expression levels being highest in BA mice. That environmental stimuli, e.g. prenatal stress or unpredictable chronic stress, alters both *Htr1a* gene expression and receptor binding has already been demonstrated in rats and mice^25, 26, 56^. In these studies, stressed animals showed a decrease of *Htr1a* expression or binding. A further key element in the serotonin system is MAOA which inactivates neuroactive monoamines such as serotonin and has been strongly implicated in panic disorders^24, 57^. In the present study, the lowest *Maoa* expression levels were found in BA mice. There is already evidence that the prevailing environment can modulate *Maoa* expression; e.g. in rats, stress during adolescence was associated with increased *Maoa* expression in the prefrontal cortex and at the same time increased anxiety-like behaviour on the EPM^27^. Altogether, these findings suggest that the effects of life history on anxiety-like behaviour are mediated, at least partly, by changes in the regulation of serotonergic pathways, especially reflected by differential *Htr1a* and *Maoa* expression.

Varying social experiences during life were also reflected by changes in hippocampal GR (*Nr3c1*) expression. Specifically, beneficial conditions during the late phase predicted elevated GR expression compared to adverse experiences in adulthood. Increased GR expression, which was associated with a dampened stress response, was already reported for the adult offspring of mothers performing high compared to low quality of maternal care^1, 23^. As the hippocampal glucocorticoid receptor system is strongly implicated in the regulation of HPA activity, increased glucocorticoid receptor expression has been suggested to mediate increased glucocorticoid feedback sensitivity^1^.

The most pronounced effects of life history were detected concerning *Avpr1a* expression. AVPR1A is a G-protein coupled receptor that binds arginine vasopressin (AVP). While there are three receptor subtypes for AVP (V1A, V1B, and V2), the V1A receptor (AVPR1A), has been suggested to play the dominant role in regulating behaviour and is implicated in the modulation of stress, anxiety, and sociability^58–60^. The finding that BA mice showed decreased levels of anxiety-like behaviour and at the same time the lowest levels of *Avpr1a* expression suggests an association between life history, anxiety-like behaviour, and *Avpr1a* expression. Interestingly, a relationship between anxiety-like behaviour and *Avp* as well as *Avpr1a* has already been demonstrated in several studies^58, 61–63^. Namely, *Avp* was found to exert anxiogenic effects and thus increased anxiety-like behaviours^63^. Similarly, it was shown that decreased *Avpr1a* expression was linked to decreased anxiety-like behaviour^58, 61, 62^. Moreover, evidence emerged that, with decreasing *Avp* expression, active coping increases^63^. Since in the present study, BA animals were provided with a situation in adulthood with which they had to cope by escaping from their offender through a tunnel-system, it is reasonable to assume that decreased *Avpr1a* expression levels were indeed linked to active coping styles. This is also underscored by the finding that the late phase rather than the early phase significantly influenced the *Avpr1a* gene expression.

Furthermore, the expression of two genes involved in the process of myelination of the brain, *Mbp* and *Gh*, was significantly influenced by life history (*Mbp*) or an early-by-late phase interaction (*Gh*), respectively. While *Mbp* expression was lowest in mice of the BA group, *Gh* expression appeared to be modulated by an early-by-late phase interaction. Only the combination of an early beneficial and a late adverse environment (BA) was associated with increased *Gh* expression compared with other combinations. Notably, it has been shown earlier that *Mbp* expression can be modulated by environmental variations, such as prenatal stress, which was linked to increased *Mbp* expression^33, 34, 64^. In addition, elevated gene expression levels of Gh have been associated with hippocampal-dependent learning but also with the exposure to an acute stressful event^65, 66^.

Since the impact of life history was most evident in terms of *Avpr1a* expression, we additionally investigated methylation in the *Avpr1a* gene in the hippocampus. The different experiences during life significantly influenced methylation at several CpG sites. Particularly experiences during the late phase left an epigenetic mark, indicating a certain degree of plasticity towards environmental stimuli, which is in line with the late phase effects on gene expression levels. Notably, mice of the BA group showed increased methylation at CpG site 13, reciprocally associated at the same time with decreased *Avpr1a* expression levels. These findings are consistent with the idea that the effect of different experiences during life on gene expression can be mediated by alterations in DNA methylation and chromatin structure, which needs to be further investigated in future studies on *Avpr1a*.

Altogether, the present findings indicate that experiences over the lifespan interact with genetic and epigenetic variations to shape the behavioural profile, particularly anxiety-like behaviour and exploratory locomotion. In addition, we show that gene expression profiles of mice were significantly altered by life history – and in particular by experiences during adulthood – with regard to the key elements of the serotonin system, *Htr1a* and *Maoa*, and furthermore *Mbp*, GR *(Nrc31)*, *Gh*, and *Avpr1a*. Moreover, also *Avpr1a* gene methylation was impacted by life history and experiences during adulthood. There is already substantial evidence for an association between *Avp* and mood as well as cognitive behaviours, making its receptors the targets of novel psychopharmacological agents^67, 68^. Thus, *Avpr1a* is a promising candidate gene for future studies on the impact of diverse experiences during life on stress, anxiety and sociability.

## Electronic supplementary material

Supplementary Information
