# Supplementary material for: Impact of varying social experiences during life history on behaviour, gene expression, and vasopressin receptor gene methylation in mice
Source: Sci Rep. 2017 Aug 18;7:8719. doi: 10.1038/s41598-017-09292-0 (PMC5562890; doi:10.1038/s41598-017-09292-0)
Supplement: Supplementary file 1 — Supplementary Information [file 41598_2017_9292_MOESM1_ESM.pdf]

Impact of varying social experiences during life history on behaviour, gene expression, and vasopressin receptor gene methylation in mice

Carina Bodden[1,2,*], Daniel van den Hove[3,4,#], Klaus-Peter Lesch[3,4,5,#], Norbert Sachser[1,2,#]

[1]Department of Behavioural Biology, University of Münster, Münster, Germany

[2]Otto Creutzfeldt Center for Cognitive and Behavioral Neuroscience, University of Münster, Münster, Germany

[3]Division of Molecular Psychiatry, Center of Mental Health, Laboratory of Translational Neuroscience, University of Würzburg, Würzburg, Germany

[4]Department of Translational Neuroscience, School for Mental Health and Neuroscience, Maastricht University, Maastricht, The Netherlands

[5]Laboratory of Psychiatric Neurobiology, Institute of Molecular Medicine, I.M. Sechenov First Moscow State Medical University, Moscow, Russia

[#]Equal contribution

*Corresponding author: carina.bodden@uni-muenster.de

Supplementary Methods.

Elevated plus-maze test: Mice were tested in the EPM on PND 75 ± 2. The plus-shaped apparatus consisted of two opposing open and closed arms (each 30 × 5 cm) with 20 cm high walls that extended from a central square (5 × 5 cm). The plus-maze apparatus was elevated 50 cm above the ground and illuminated by a light bulb (150 lx). After spending 1 min in an empty cage, each mouse was individually placed on the central platform facing a closed arm and allowed to freely explore the apparatus.

Dark-light test: At the age of 76 ± 2 days mice were tested in the DL. The apparatus consisted of a modified Makrolon cage type III, which was separated into two compartments by a partition including a sliding door. The dark compartment (17 × 27 × 16 cm) was painted black, had an opaque lid, and was unlit, whereas the light compartment (28 × 27 × 16 cm) had transparent walls, no lid, and was illuminated by overhead lighting (570 lx). Each mouse was placed inside the dark compartment with the lid and sliding door closed and remained there for 1 min before the sliding door was opened and the mouse could freely explore the apparatus.

Open-field test: The OF was performed on PND 77 ± 2. The OF consisted of a white square arena (80 × 80 × 42 cm) with surrounding walls and was illuminated by an overhead bulb (600 lx). Each mouse was placed individually inside a cylinder (11 cm diameter, 20 cm high) standing in one corner of the OF apparatus. After 1 min the cylinder was lifted and the mouse was allowed to freely explore the arena. The parameters analysed were the time spent in the centre of the arena (defined as the area of the OF being located at least 20 cm distant from the walls) to measure anxiety-like behaviour and the distance travelled for assessing exploratory locomotion.

Gene expression analysis: The tissue was homogenized using 300 µl Qiazol (Qiagen, Hilden, Germany) and metal beads (Qiagen) in a TissueLyser (1 min, 20 Hz, 4°C, Qiagen). Subsequently, 60 µl chloroform was added and samples were centrifuged (5 min, 4°C, 12.000 rpm). The aqueous phase was then mixed with 1.5 vol. ethanol (95-100%). From this point on, the miRNeasy Mini Kit (Qiagen) was used as instructed by the manufacturer. The quality of the extracted RNA was analysed using NanoDrop spectrophotometer (Thermo Fisher Scientific Inc., Wilmington, USA) and Experion™ Automated Electrophoresis System (Bio-Rad, Munich, Germany). cDNA synthesis was performed using the iScript™cDNA Synthesis Kit according to the manufacturer's protocol (Bio-Rad).

DNA methylation analysis: Genomic DNA from the hippocampus was isolated using phenol/chloroform/isoamyl alcohol extraction. All centrifugation steps were performed at 14,000 rpm. The frozen, crushed tissue was homogenized using 300 µl 0.5%-SDS extraction buffer and metal beads

(Qiagen) in a TissueLyser (1 min, 25 Hz, 4°C, Qiagen). Subsequently, 200 µl 0.5% SDS extraction buffer and 50 µl 10 mg/µl-proteinase K was added and incubated overnight at 55°C. Afterwards, samples were incubated with 50 µl 10 mg/µl-RNAse A at 37°C for 1 h, followed by the addition of 700µl of phenol/chloroform/isoamyl alcohol solution (25:24:1). Samples were mixed thoroughly, whereupon phases were separated by centrifuging for 5 min at 14000 rpm at RT in Maxtract high density tubes (Qiagen). The aqueous phase was mixed with 700 µl chloroform/isoamyl alcohol (24:1) and phases were once again separated as described above. DNA precipitation was evoked by incubating the samples with 50 µl sodium acetate (3M, ph 5.2) and 1000 µl ice-cold ethanol (95-100%) for 10 min at -20°C, followed by 20 min centrifugation at 4°C. The DNA pellet was washed with 500 µl cold 80%-ethanol following the same procedure as for the precipitation. After the pellet was air-dried for 20 to 30 min at RT, resuspension followed using 50 µl 1xTE. The quality of the extracted DNA was analysed using NanoDrop spectrophotometer (Thermo Fisher Scientific Inc.).

Supplementary Table S1. Primer sequences for gene expression analyses using qRT-PCR.

Gene		Primer	Sequence	Product size (bp)
Htr1a	*Serotonin receptor 1a*	F	AACCAGTTTTGTGTCCTCTCA	115
		R	AGCACCTAAATAATTTTCTTCTCTG	
Htr2a	*Serotonin receptor 2a*	F	AACCCCATTCACCATAGCCG	107
		R	CCGAAGACTGGGATTGGCAT	
Ar	*Androgen receptor*	F	CCAGTCCCAATTGTGTCAAA	91
		R	TCCCTGGTACTGTCCAAACG	
Avpr1a	*Arginine vasopressin receptor 1a*	F	CAATTTCGTTTGGACCGATT	91
		R	GTAGATCCACGGGTTGCAG	
Bdnf Ex I	*Brain-derived neurotrophic factor, exon 1*	F	AGTCTCCAGGACAGCAAAGC	94
		R	TGCAACCGAAGTATGAAATAACC	
Bdnf Ex IV	*Brain-derived neurotrophic factor, exon 4*	F	GATCCGAGAGCTTTGTGTGG	76
		R	AACCATAGTAAGGAAAAGGATGGTC	
Crhr1	*Corticotropin releasing hormone receptor 1*	F	GCCTTTTTCTACGGTGTCCG	115
		R	CGTTGAGAATCTCCTGGCACT	
Gdi2	*Guanosine diphosphate dissociation inhibitor 2*	F	GTCAGAATTGGTTGGTTCTGTTC	126
		R	AGCTCTTGGATCACACAATCG	
Gh	*Growth hormone*	F	CACCTCGGACCGTGTCTATG	105
		R	TTGAGGATCTGCCCAACACG	
Maoa	*Monoamine oxidase A*	F	TCGGGAGAATTTTACCCAAACCA	166
		R	AACTCTATCCCGGGCTTCCA	
Mbp	*Myelin basic protein*	F	CTCCCTGCCCCAGAAGTC	95
		R	GAGGTGGTGTTCGAGGTGTC	
Npy	*Neuropeptide Y*	F	CCGCTCTGCGACACTACAT	68
		R	TGTCTCAGGGCTGGATCTCT	
Nr3c1	*Glucocorticoid receptor*	F	AACTGGAATAGGTGCCAAGG	125
		R	GAACTCACATCTGGTCTCATTCC	
Oxtr	*Oxytocin receptor*	F	TCTTCGTGCAGATGTGGAGC	128
		R	GCCCGTGAAGAGCATGTAGA	
Snip1	*Smad nuclear interacting protein 1*	F	CGTGGCTTCTACCAACAGG	129
		R	CAAAGCTAAAGAAAAAGACCAGATG	
Tbp	*TATA-binding protein*	F+R	QuantiTect Primer Assay (QT00198443)	114

F = Forward, R = Reverse, bp = base pairs

Supplementary Table S2. Sequences analysed for DNA methylation within the *Avpr1a* gene in the hippocampus using pyrosequencing.

Assay	CpG site	Sequence to analyse	Amplicon length (bp)
Mm_Avpr1a_01_PM	7-11	TTATTTAT**Y**GTTTT**Y**G**Y**GGGT**Y**GGATTGGTTGTGT**Y**GGGT	91
Mm_Avpr1a_02_PM	2-6	**Y**GTTGTAT**Y**GTA**Y**GTTA**Y**GTAAGATATTT**Y**GT	104
Mm_Avpr1a_04_PM	12-16	**Y**GGGTGGTATA**Y**GG**Y**GATGTAG**Y**GGTTAGT**Y**GT	191
Mm_Avpr1a_TF	1	GG**Y**GTTGTTTTTGAAGAAGGAATATATAT	103

bp = base pairs

Supplementary Table S3. Effects of life history on anxiety-like and exploratory behaviour, gene expression and DNA methylation. Data analysis was performed using General Linear Models. The statistical analysis is summarized with respect to effects of life history, early phase, late phase, and early-by-late phase interaction. CNRQ = Calibrated Normalized Relative Quantities.

	Life History		Early phase		Late phase		Early x Late phase	
	F	p-value	F	p-value	F	p-value	F	p-value
**Elevated plus-maze**								
Time on open arms (%)	2.368	0.063	3.915	0.054	2.731	0.105	0.753	0.390
Entries into open arms (%)	1.927	0.118	4.398	**0.042**	0.014	0.907	0.817	0.371
Sum of entries (#)	2.783	**0.035**	0.005	0.943	10.313	**0.002**	0.116	0.735
**Dark-light**								
Entries into light compartment (#)	2.868	**0.031**	0.941	0.337	8.912	**0.005**	0.269	0.606
Time in light compartment (s)	2.245	0.075	1.314	0.258	3.616	0.063	1.452	0.234
Latency to light compartment (s)	0.641	0.635	1.148	0.290	1.274	0.265	0.118	0.733
**Open field**								
Time in centre (s)	2.632	**0.043**	0.075	0.786	3.236	0.079	0.002	0.968
Distance (m)	3.293	**0.017**	0.844	0.363	5.409	**0.025**	2.134	0.151
**Gene expression in the hippocampus [CNRQ]**								
Hr1a	3.533	**0.012**	2.088	0.156	2.314	0.135	2.849	0.098
Htr2a	0.636	0.639	0.424	0.518	0.342	0.562	0.326	0.571
Ar	0.730	0.575	<0.001	0.997	2.475	0.123	<0.001	0.999
Avpr1a	9.501	**<0.001**	2.556	0.117	24.323	**<0,001**	1.119	0.296
Bdnf I	0.090	0.985	0.206	0.652	0.072	0.790	0.051	0.822
Bdnf IV	0.869	0.488	0.371	0.545	2.643	0.111	0.117	0.734
Crhr1	0.719	0.582	1.250	0.270	1.463	0.233	0.029	0.866
Gh	1.931	0.118	2.101	0.154	1.545	0.221	5.394	**0.025**
Maoa	3.196	**0.020**	0.650	0.425	6.514	**0.014**	3.418	0.071
Mbp	2.832	**0.033**	0.453	0.504	6.769	**0.013**	2.481	0.122
Npy	0.664	0.620	0.849	0.362	0.830	0.367	0.641	0.428
Nr3c1	2.053	0.099	0.015	0.902	6.658	**0.013**	0.403	0.529
Oxtr	0.639	0.637	0.086	0.770	1.410	0.241	0.312	0.579
**Gene expression in the amygdala [CNRQ]**								
Hr1a	0.688	0.603	0.028	0.868	2.405	0.128	0.123	0.727
Htr2a	0.069	0.991	<0.001	0.999	0.005	0.946	0.138	0.712
Ar	0.652	0.628	0.112	0.740	1.685	0.201	0.143	0.707
Avpr1a	0.957	0.438	0.007	0.932	1.455	0.234	0.904	0.347
Bdnf I	0.702	0.594	1.086	0.303	1.625	0.209	0.025	0.876
Bdnf IV	0.611	0.657	0.879	0.354	1.070	0.307	0.001	0.978
Crhr1	1.007	0.412	0.849	0.362	2.175	0.147	0.104	0.748
Gh	0.957	0.438	1.280	0.265	0.002	0.964	0.027	0.869
Maoa	1.218	0.313	1.581	0.215	2.927	0.094	0.023	0.881
Mbp	1.078	0.376	0.031	0.861	2.195	0.146	0.069	0.794
Npy	1.006	0.412	0.014	0.907	4.248	**0.045**	0.327	0.570
Nr3c1	1.086	0.372	0.241	0.626	2.436	0.126	0.207	0.651
Oxtr	1.165	0.336	0.217	0.644	3.374	0.073	0.637	0.429
**Avpr1a DNA methylation in the hippocampus [%]**								
CpG site 1	0.830	0.512	0.614	0.438	1.161	0.287	0.133	0.718
CpG site 2	0.823	0.517	0.053	0.820	2.031	0.162	0.327	0.571
CpG site 3	1.210	0.318	0.237	0.629	3.477	0.070	0.913	0.345
CpG site 4	0.575	0.682	0.439	0.511	0.896	0.350	0.463	0.500
CpG site 5	0.690	0.602	0.130	0.721	1.779	0.190	0.656	0.423
CpG site 6	1.625	0.183	0.057	0.813	4.758	**0.035**	1.305	0.260
CpG site 7	0.489	0.743	0.241	0.626	0.939	0.338	0.615	0.437
CpG site 8	1.476	0.222	0.140	0.710	4.052	**0.050**	0.249	0.620
CpG site 9	0.499	0.737	0.228	0.635	0.177	0.676	0.128	0.723
CpG site 10	1.164	0.337	1.386	0.246	0.455	0.504	0.001	0.975
CpG site 11	0.903	0.469	1.668	0.203	<0.001	0.993	0.203	0.654
CpG site 12	0.918	0.460	0.763	0.387	0.881	0.353	1.685	0.201
CpG site 13	2.957	**0.028**	2.175	0.148	6.768	**0.013**	1.720	0.197
CpG site 14	0.339	0.851	0.329	0.569	0.147	0.703	0.392	0.535
CpG site 15	0.726	0.578	1.776	0.190	0.097	0.757	0.027	0.871
CpG site 16	0.817	0.520	0.043	0.837	2.522	0.120	0.139	0.711

Supplementary Table S4. Effects of life history on anxiety-like and exploratory behaviour, gene expression and DNA methylation. Data are given as means of experimental group ± standard error of the mean (s.e.m.). CNRQ = Calibrated Normalized Relative Quantities.

	AA	AB	BA	BB	SH
	Mean ± s.e.m.	Mean ± s.e.m.	Mean ± s.e.m.	Mean ± s.e.m.	Mean ± s.e.m.
**Elevated plus-maze test**					
Time on open arms (%)	24.45 ± 3.59	22.11 ± 2.38	32.93 ± 2.16	25.42 ± 3.49	31.67 ± 3.42
Entries into open arms (%)	36.51 ± 3.62	39.70 ± 2.70	45.90 ± 2.29	43.44 ± 3.70	46.24 ± 2.74
Sum of entries (#)	28.77 ± 2.86	23.42 ± 1.06	29.54 ± 1.37	22.92 ± 1.45	26.62 ± 1.54
**Dark-light test**					
Entries into light compartment (#)	10.77 ± 1.50	7.58 ± 1.30	12.69 ± 1.11	8.17 ± 1.20	9.38 ± 0.90
Time in light compartment (s)	63.81 ± 12.35	56.41 ± 10.94	88.78 ± 10.15	55.78 ± 8.30	83.79 ± 9.48
Latency to light compartment (s)	33.95 ± 20.44	46.99 ± 21.59	10.46 ± 2.19	34.91 ± 15.28	30.90 ± 16.37
**Open field test**					
Time in centre (s)	12.13 ± 1.84	8.52 ± 1.36	11.52 ± 2.46	8.06 ± 1.94	16.44 ± 2.44
Distance (m)	35.11 ± 1.46	28.74 ± 1.62	34.20 ± 1.72	32.74 ± 1.92	36.37 ± 1.27
**Gene expression in the hippocampus [CNRQ]**					
Hr1a	1.00 ± 0.02	1.00 ± 0.01	1.11 ± 0.07	0.99 ± 0.03	0.94 ± 0.02
Htr2a	1.02 ± 0.06	1.02 ± 0.06	1.11 ± 0.10	1.03 ± 0.04	0.97 ± 0.04
Ar	0.98 ± 0.05	1.07 ± 0.05	0.98 ± 0.08	1.07 ± 0.04	1.01 ± 0.03
Avpr1a	0.94 ± 0.06	1.16 ± 0.05	0.78 ± 0.06	1.12 ± 0.05	1.15 ± 0.04
Bdnf I	1.00 ± 0.06	1.01 ± 0.06	1.02 ± 0.06	1.05 ± 0.06	1.01 ± 0.04
Bdnf IV	0.96 ± 0.07	1.04 ± 0.05	0.98 ± 0.06	1.10 ± 0.07	1.02 ± 0.04
Crhr1	1.04 ± 0.05	1.11 ± 0.06	0.96 ± 0.09	1.05 ± 0.05	1.00 ± 0.07
Gh	3.11 ± 1.67	8.87 ± 2.61	23.24 ± 10.28	4.21 ± 3.53	32.35 ± 16.82
Maoa	1.02 ± 0.02	1.04 ± 0.02	0.93 ± 0.05	1.07 ± 0.03	0.98 ± 0.02
Mbp	1.03 ± 0.06	1.09 ± 0.05	0.90 ± 0.06	1.14 ± 0.05	0.97 ± 0.05
Npy	0.97 ± 0.03	1.03 ± 0.04	1.03 ± 0.03	1.03 ± 0.03	0.99 ± 0.04
Nr3c1	0.97 ± 0.06	1.08 ± 0.05	0.92 ± 0.08	1.11 ± 0.05	1.06 ± 0.04
Oxtr	0.99 ± 0.02	1.11 ± 0.06	1.01 ± 0.10	1.05 ± 0.09	1.00 ± 0.04
**Gene expression in the amygdala [CNRQ]**					
Hr1a	0.97 ± 0.05	1.09 ± 0.05	0.98 ± 0.08	1.06 ± 0.07	1.02 ± 0.05
Htr2a	1.06 ± 0.11	1.12 ± 0.14	1.11 ± 0.14	1.07 ± 0.16	1.14 ± 0.12
Ar	0.98 ± 0.09	1.16 ± 0.09	0.99 ± 0.11	1.08 ± 0.13	1.12 ± 0.08
Avpr1a	0.96 ± 0.05	1.14 ± 0.08	1.04 ± 0.11	1.07 ± 0.09	0.97 ± 0.05
Bdnf I	1.02 ± 0.06	1.13 ± 0.08	0.95 ± 0.08	1.03 ± 0.10	1.06 ± 0.08
Bdnf IV	1.03 ± 0.07	1.13 ± 0.08	0.95 ± 0.10	1.04 ± 0.11	1.11 ± 0.09
Crhr1	1.00 ± 0.08	1.17 ± 0.08	0.94 ± 0.10	1.05 ± 0.10	1.13 ± 0.09
Gh	3.36 ± 1.90	3.80 ± 1.19	8.07 ± 4.19	7.30 ± 5.70	5.04 ± 2.15
Maoa	1.01 ± 0.05	1.09 ± 0.05	0.93 ± 0.06	1.03 ± 0.06	1.05 ± 0.05
Mbp	0.96 ± 0.06	1.05 ± 0.08	0.95 ± 0.08	1.09 ± 0.09	1.12 ± 0.06
Npy	0.97 ± 0.02	1.04 ± 0.02	0.99 ± 0.02	1.03 ± 0.03	1.00 ± 0.03
Nr3c1	0.96 ± 0.08	1.16 ± 0.10	0.96 ± 0.10	1.06 ± 0.12	1.16 ± 0.10
Oxtr	0.97 ± 0.10	1.30 ± 0.10	1.01 ± 0.13	1.14 ± 0.17	1.16 ± 0.11
**Avpr1a DNA methylation in the hippocampus [%]**					
CpG site 1	4.39 ± 0.26	4.54 ± 0.19	4.15 ± 0.20	4.45 ± 0.15	4.63 ± 0.14
CpG site 2	5.08 ± 0.30	4.45 ± 0.38	4.82 ± 0.29	4.56 ± 0.27	5.08 ± 0.32
CpG site 3	5.48 ± 0.19	4.77 ± 0.33	5.36 ± 0.27	5.13 ± 0.25	5.58 ± 0.34
CpG site 4	6.10 ± 0.33	5.61 ± 0.30	6.09 ± 0.27	6.01 ± 0.23	6.30 ± 0.33
CpG site 5	6.24 ± 0.17	5.79 ± 0.26	5.99 ± 0.21	5.89 ± 0.19	6.19 ± 0.28
CpG site 6	8.74 ± 0.20	8.03 ± 0.17	8.54 ± 0.22	8.32 ± 0.22	8.66 ± 0.22
CpG site 7	2.24 ± 0.04	2.23 ± 0.07	2.37 ± 0.17	2.20 ± 0.08	2.21 ± 0.09
CpG site 8	2.05 ± 0.09	2.34 ± 0.11	2.06 ± 0.13	2.24 ± 0.13	2.28 ± 0.09
CpG site 9	0.81 ± 0.06	0.75 ± 0.05	0.81 ± 0.12	0.81 ± 0.03	0.89 ± 0.08
CpG site 10	1.61 ± 0.10	1.69 ± 0.11	1.48 ± 0.14	1.55 ± 0.10	1.36 ± 0.13
CpG site 11	2.13 ± 0.10	2.08 ± 0.11	1.94 ± 0.08	1.99 ± 0.13	2.19 ± 0.10
CpG site 12	0.74 ± 0.12	0.78 ± 0.09	0.97 ± 0.09	0.73 ± 0.12	0.85 ± 0.08
CpG site 13	1.01 ± 0.06	0.87 ± 0.09	1.32 ± 0.17	0.89 ± 0.11	1.13 ± 0.09
CpG site 14	0.64 ± 0.11	0.67 ± 0.10	0.79 ± 0.15	0.66 ± 0.14	0.79 ± 0.13
CpG site 15	1.39 ± 0.08	1.36 ± 0.08	1.19 ± 0.28	1.11 ± 0.17	1.39 ± 0.07
CpG site 16	1.52 ± 0.11	1.38 ± 0.14	1.54 ± 0.12	1.31 ± 0.09	1.39 ± 0.07

Supplementary Data S1. Raw data resulting from behavioural testing in the Elevated plus-maze (EPM), Dark-light (DL), and Open-field (OF) test. Data are separately presented for each parameter that was analysed.

		EPM			DL			OF	
**Animal ID**	**Life history**	**Entries into open arms [%]**	**Time in open arms [%]**	**Sum of entries [#]**	**Entries into light [#]**	**Time in light [s]**	**Latency to light [s]**	**Time in center [s]**	**Total distance [m]**
597	AA	44.0	19.4	25	10	50.9	2.7	9.9	29.93
603	AA	49.0	37.5	49	16	103.6	6.3	7.6	42.92
619	AA	25.0	17.4	32	12	52.7	7.7	14.0	39.94
722	AA	33.3	7.3	18	14	55.3	10.0	17.6	33.27
736	AA	33.3	26.6	30	7	43.8	7.5	4.2	29.53
743	AA	29.4	16.4	34	8	19.3	98.8	20.9	38.53
744	AA	41.9	20.2	31	11	69.1	27.4	6.3	32.73
764	AA	42.9	43.1	28	1	2.5	263.2	5.0	25.84
768	AA	44.0	22.8	25	7	43.4	8.9	9.9	35.13
834	AA	0.0	0.0	4	16	148.1	0.8	9.0	32.06
862	AA	41.2	30.7	34	17	77.2	0.8	27.3	34.48
873	AA	45.7	39.9	35	18	142.4	1.1	12.7	39.55
976	AA	44.8	36.7	29	3	21.2	6.2	13.3	42.56
602	AB	45.2	28.2	31	10	85.6	1.9	18.6	40.95
615	AB	51.9	33.7	27	1	2.3	211.0	5.6	26.23
617	AB	48.0	24.4	25	16	108.8	1.4	4.4	23.13
635	AB	33.3	6.9	18	11	100.6	0.7	5.3	27.69
721	AB	22.7	10.9	22	3	5.6	2.3	2.1	30.96
732	AB	33.3	12.8	18	6	50.7	166.5	12.9	23.53
734	AB	45.8	24.9	24	4	28.8	122.5	12.6	24.34
738	AB	53.8	30.2	26	4	22.6	5.2	10.9	29.75
759	AB	33.3	21.5	24	13	94.5	11.2	10.4	36.31
770	AB	40.9	19.4	22	5	30.1	12.4	4.3	21.98
772	AB	33.3	28.4	21	8	68.1	18.3	6.8	29.33
871	AB	34.8	24.1	23	10	79.2	10.5	8.3	30.63
605	BA	42.3	27.2	26	7	83.1	21.5	13.4	30.58
623	BA	64.3	44.5	28	17	92.0	10.9	11.9	34.36
627	BA	48.1	26.9	27	8	51.7	23.1	4.6	32.79
728	BA	29.2	18.4	24	17	97.6	1.1	5.2	24.34
755	BA	46.7	34.0	30	12	102.6	5.8	3.6	32.15
766	BA	51.9	28.7	27	8	18.0	7.2	12.0	29.72
842	BA	46.7	34.4	30	13	103.8	11.4	9.1	41.79
847	BA	41.2	24.2	34	15	87.2	0.8	18.3	33.44
852	BA	40.7	35.1	27	10	61.5	8.1	11.6	34.27
865	BA	51.2	41.1	41	19	134.5	1.7	37.0	48.92
887	BA	48.5	38.1	33	9	50.3	23.4	11.1	32.18
947	BA	38.2	31.9	34	16	122.1	11.0	2.3	39.67
957	BA	47.8	43.7	23	14	149.7	10.0	9.6	30.39
610	BB	63.2	49.9	19	17	114.9	2.1	0.5	26.23
626	BB	42.3	33.1	26	10	43.2	1.2	9.7	33.29
719	BB	38.5	9.1	13	12	49.5	1.7	2.2	38.87
751	BB	33.3	17.6	27	4	34.1	161.3	9.2	28.31
757	BB	42.9	16.9	21	9	47.6	89.0	4.3	31.33
836	BB	32.1	18.3	28	8	49.1	2.3	3.1	26.16
846	BB	55.6	32.0	18	4	39.4	3.5	9.6	28.90
850	BB	27.8	12.3	18	4	50.4	24.5	2.5	24.77
866	BB	48.3	22.9	29	4	40.9	27.3	24.4	37.34
867	BB	24.0	20.2	25	9	50.3	0.9	11.4	47.95
885	BB	59.3	33.9	27	5	33.0	100.5	5.1	35.91
958	BB	54.2	38.8	24	12	117.0	4.6	14.7	33.86
854	SH	29.4	3.4	17	6	26.0	194.9	13.9	35.55
875	SH	44.4	41.6	27	11	71.8	42.8	14.8	33.18
876	SH	50.0	31.5	30	13	75.2	2.4	3.3	30.80
879	SH	40.9	28.3	22	6	57.1	118.3	10.6	34.58
898	SH	40.0	31.7	35	15	129.5	0.6	14.8	46.30
931	SH	30.0	15.1	20	6	40.6	4.4	15.1	39.34
933	SH	55.6	31.1	27	7	83.5	14.1	24.2	33.65
937	SH	56.0	43.7	25	11	123.8	1.8	40.6	41.43
939	SH	46.2	32.1	26	13	101.7	0.6	14.3	32.92
940	SH	63.6	51.8	22	6	80.8	8.7	9.5	30.67
969	SH	51.7	27.3	29	12	116.2	0.7	15.4	38.98
970	SH	50.0	39.6	36	8	128.3	5.0	18.9	39.92
971	SH	43.3	34.4	30	8	54.8	7.4	18.3	35.51

Supplementary Data S2. Raw data resulting from gene expression analyses in the hippocampus. Data are separately presented for each gene that was analysed. CNRQ = Calibrated Normalized Relative Quantities.

		Relative gene expression in the hippocampus [CNRQ]												
**Animal ID**	**Life history**	***Htr1a***	***Htr2a***	***Ar***	***Avpr1a***	***Bdnf I***	***Bdnf IV***	***Crhr1***	***Gh***	***Maoa***	***Mbp***	***Npy***	***Nr3c1***	***Oxtr***
597	AA	0.94404	1.08339	1.11012	0.89260	1.08917	1.09072	1.36623	0.79040	1.07647	1.23250	1.00261	1.33005	0.96285
603	AA	1.02000	0.46631	0.57537	1.49400	0.70397	0.54797	0.87733	0.14680	0.93542	0.80575	1.16680	0.54641	1.00585
619	AA	1.02427	0.96738	0.95301	1.12700	0.86698	0.89885	1.07343	1.51400	1.07431	1.11420	1.11378	0.94290	1.07389
722	AA	0.96820	0.94060	1.05936	0.93790	1.23684	1.31125	0.89894	21.02000	1.05101	0.70580	1.08166	0.96129	0.97492
736	AA	1.01210	1.14602	1.11816	1.02900	0.95225	1.19034	1.22048	0.00007	1.12894	1.21113	0.81941	1.12780	1.03023
743	AA	1.01496	1.23060	1.09132	0.85330	1.10232	0.95820	0.99601	0.00007	0.97481	1.04078	0.97573	1.00547	1.01933
744	AA	0.90978	1.05741	0.96053	1.08300	1.11671	1.12402	0.79641	0.23680	0.93987	0.73759	1.02047	1.10249	0.92213
764	AA	0.79404	1.03276	0.96042	0.97200	0.91965	0.99836	1.15876	1.11600	0.99001	1.30291	0.86559	1.01974	0.83024
768	AA	1.05054	1.06751	1.10865	0.75340	1.18939	1.03661	0.94227	1.74100	1.02141	1.29602	0.86557	1.03542	0.92364
834	AA	1.09114	0.74526	0.74079	0.68170	0.67479	0.56774	0.91451	0.04316	0.93895	0.94896	0.91983	0.63414	0.94157
862	AA	0.98555	1.36329	1.14102	0.89720	1.41102	1.20003	1.14745	3.10100	1.13996	1.28102	1.00353	1.17046	1.06195
873	AA	1.05687	1.10587	1.08055	0.85980	0.86770	0.84023	1.17756	0.77900	0.97660	0.99432	0.81072	0.90934	1.06189
976	AA	1.07053	1.06244	0.84342	0.57870	0.92864	0.77207	0.96656	9.94800	0.98640	0.71166	0.96289	0.76252	1.03383
602	AB	1.03056	1.05039	1.13984	1.41800	1.27912	1.19302	0.85376	0.00007	1.04436	1.21439	1.09008	1.11232	1.14266
615	AB	1.00265	0.96392	1.18830	1.45200	0.92209	0.95712	1.07154	23.14000	1.05727	1.01553	1.01299	1.05542	1.19579
617	AB	1.00906	1.17056	1.15291	1.14600	1.25607	1.30953	1.25117	1.75000	1.04263	1.33653	1.16173	1.14970	1.07233
635	AB	1.02898	0.64669	0.72156	1.31900	0.75034	0.72784	1.36000	15.69000	1.11578	1.08189	1.16031	0.78086	1.23169
721	AB	0.98600	0.80914	0.83001	0.81750	0.79003	0.85623	0.80626	21.68000	0.97381	0.77801	1.18075	0.81039	0.99451
732	AB	1.02932	1.09880	1.15154	1.19000	0.82256	0.97988	1.33097	8.52100	1.10696	1.04835	0.92767	1.26401	1.09792
734	AB	1.00066	1.32333	1.20514	0.98141	1.31600	1.24323	0.92878	1.00500	1.00905	0.86091	1.08571	1.07876	1.15567
738	AB	0.98291	0.97167	1.11231	1.21700	0.88026	1.07996	1.11338	12.70000	0.92894	1.34130	0.77842	1.17788	0.97353
759	AB	0.98158	1.02145	0.99989	0.99420	0.91544	1.08416	0.87150	18.47000	1.08110	1.26235	1.01675	0.97457	0.91979
770	AB	0.94078	0.78978	0.99036	1.15200	0.92080	1.00707	1.10819	3.28800	0.95189	1.03313	0.94241	1.00314	0.89038
772	AB	0.99776	0.98139	1.08238	1.09800	0.89115	0.99142	1.24213	0.06430	1.04139	0.97503	0.88965	1.19885	1.03871
871	AB	1.03211	1.40894	1.24560	1.08200	1.34098	1.10323	1.36430	0.13360	1.13799	1.12461	1.06637	1.36839	1.65971
605	BA	0.67766	0.44749	0.31135	0.36990	0.74850	0.60716	0.26519	13.53000	0.47391	0.48904	0.99250	0.33641	0.22303
623	BA	1.13064	1.14879	1.01195	0.75850	1.11060	0.95346	0.85514		0.98273	0.86694	1.12534	0.91275	1.11853
627	BA	0.94756	0.52939	0.56103	0.55650	0.64385	0.49060	0.50143	0.06948	0.67723	0.64501	1.17622	0.47321	0.68101
728	BA	1.19374	0.96603	1.06084	0.79930	1.18743	1.03520	0.78843	3.22500	0.98694	0.67494	0.93075	0.91681	1.04709
755	BA	0.93249	1.04490	1.00962	0.77670	1.06489	1.17621	1.11508	17.63000	0.97363	0.98061	1.00239	0.94790	0.85355
766	BA	1.19062	1.56507	1.14939	0.89340	0.83845	1.00676	1.10984	10.81000	0.99520	1.15365	0.83226	0.95625	1.04091
842	BA	1.00807	1.06443	0.98669	0.94790	1.08529	1.02384	1.32368	0.97380	0.96854	0.75589	0.97773	0.91677	0.94362
847	BA	1.31992	1.32863	1.21911	0.79050	1.08112	1.01764	1.13428	111.30000	1.02064	0.98697	0.94441	1.02512	1.47780
852	BA	1.33641	1.36071	1.15990	0.70480	1.32809	1.07928	1.13172	5.35700	1.07672	1.09890	1.04973	1.19051	1.35544
865	BA	1.23777	1.37914	1.17568	0.77770	1.16008	1.19369	1.06718	35.46000	1.04988	1.06324	1.17006	1.10981	1.07718
887	BA	0.90504	1.03581	0.98262	1.16500	1.09251	1.06432	0.92376	57.11000	0.91541	0.94252	0.99722	1.13522	0.80506
947	BA	1.49075	1.40737	1.13554	0.87200	0.87791	1.12392	1.27915	0.14230	1.05742	1.12163	1.11846	1.11916	1.46161
957	BA													
610	BB	0.86425	1.03678	1.05169	1.05100	1.12929	1.07735	0.91330	0.16220	1.11282	1.31279	0.97393	1.10960	1.03286
626	BB	1.07343	0.96937	1.05318	1.35100	0.81316	0.94094	0.91609	5.17400	1.11424	1.06032	0.89878	0.99273	1.09236
719	BB	0.99838	1.00196	1.21934	0.89540	0.94678	1.10293	1.10000	0.56200	1.15508	1.37605	1.10160	1.20746	0.97663
751	BB	1.04663	1.20811	1.21101	1.14400	1.23622	1.31447	1.12395	0.17100	1.14238	1.22579	0.88161	1.36642	1.04875
757	BB	0.86535	0.85480	0.82835	1.16400	0.95419	1.02785	1.02765	0.04312	1.01033	0.88983	0.89194	1.00665	0.78272
836	BB													
846	BB	1.03920	0.96696	1.09686	1.34400	0.86584	0.96121	1.26000	0.00007	1.23227	1.18030	1.03656	1.11263	1.13466
850	BB	1.07251	0.95549	1.09422	0.97060	0.77381	0.83085	1.08722	0.53850	1.07575	1.15339	0.99890	1.09183	1.22667
866	BB	0.85459	0.83006	0.84062	1.10200	1.15515	1.01586	0.78889	0.43120	0.96413	0.93991	1.11762	0.89870	0.93233
867	BB	1.17388	1.29230	1.31510	1.14400	1.32488	1.11027	1.40637	39.18000	1.15213	1.40188	1.10847	1.38610	1.79712
885	BB	0.95344	1.00059	1.03500	0.87520	1.03806	1.02330	1.05610	0.04247	0.88844	1.01777	1.07248	1.00966	0.61112
958	BB	0.97758	1.16202	1.00933	1.33200	1.28811	1.73590	0.83889	0.03447	0.97633	1.00352	1.24862	1.04658	0.94341
854	SH	0.82517	0.90154	0.97024	0.92320	0.94921	0.87249	0.88669	0.03922	0.90803	0.94305	0.88020	0.92020	0.90582
875	SH	0.90805	0.81090	0.76759	0.99240	1.10708	0.98555	0.63004	5.23700	0.89498	0.64480	1.05797	0.86011	0.76561
876	SH	0.88015	0.89489	0.96786	1.06500	0.84398	0.86521	1.16703	0.61310	0.87282	1.23549	1.01207	0.99916	0.92625
879	SH	0.98740	0.97878	1.10859	1.07700	1.07755	1.03149	1.16284	22.26000	1.03447	0.95128	0.90012	1.05493	1.11304
898	SH	0.93254	0.83258	0.92925	1.13700	1.17100	1.26641	0.74519	1.74900	0.89876	0.86706	1.24205	1.03566	0.80433
931	SH	1.03047	1.12118	1.03761	1.08000	0.94507	1.04055	1.01377	0.07903	1.05153	1.19621	0.99150	1.10031	1.04423
933	SH	0.92117	1.02802	1.15582	1.25700	1.01135	0.96094	1.66112	0.90600	1.12176	1.19246	0.84255	1.35588	1.08734
937	SH	0.90328	0.84074	0.87094	1.06000	1.09632	1.10812	0.85594	7.21600	0.95472	0.82475	1.13375	0.87017	0.86020
939	SH	0.97896	1.08213	1.10198	1.36500	0.88001	0.99966	0.95691	0.51710	1.05582	0.86003	0.79728	1.20313	1.14103
940	SH	0.94281	1.23583	1.04710	1.44200	1.27903	1.33211	0.91662	196.60000	0.95377	0.96135	1.15020	1.22202	0.95482
969	SH	0.89152	1.01318	1.10740	1.23100	1.12543	1.01865	1.13588	122.50000	0.95693	0.80959	0.86837	1.13667	1.16289
970	SH	1.00983	0.84715	1.04551	1.17300	0.77007	0.82086	0.89730	59.24000	1.01399	1.06528	1.05944	0.93112	1.04133
971	SH	1.00306	0.95947	1.07993	1.18800	0.92685	1.02059	0.97781	3.56100	1.03067	1.03114	0.90886	1.04422	1.13276

Supplementary Data S3. Raw data resulting from gene expression analyses in the amygdala. Data are separately presented for each gene that was analysed. CNRQ = Calibrated Normalized Relative Quantities.

		Relative gene expression in the amygdala [CNRQ]												
Animal ID	Life history	*Htr1a*	*Htr2a*	*Ar*	*Avpr1a*	*Bdnf I*	*Bdnf IV*	*Crhr1*	*Gh*	*Maoa*	*Mbp*	*Npy*	*Nr3c1*	*Oxtr*
597	AA	0.78505	0.60751	0.65877	0.80561	0.85652	0.82675	0.88027	0.38719	0.91453	0.80946	0.97565	0.68001	0.94962
603	AA	0.68153	0.57792	0.46047	0.87524	0.54687	0.49574	0.58869		0.75091	0.94827	0.91557	0.48948	0.57012
619	AA	1.01672	1.21676	1.29225	0.86000	1.42856	1.46094	1.15874	3.43878	1.03011	0.71838	0.92078	1.11445	1.03269
722	AA	1.06286	1.28034	1.16008	0.93781	1.09353	0.99955	1.04663	21.77231	1.08592	1.13315	1.16580	1.02067	0.83154
736	AA	0.97062	1.34194	1.33850	0.86729	1.19628	1.45058	1.14295		1.16219	1.00897	0.92635	1.32239	1.26370
743	AA	0.71346	0.69396	0.42236	0.90861	0.91747	0.98144	0.40927	0.02512	0.64878	0.50647	0.78689	0.56824	0.33218
744	AA	1.21134	1.87871	1.26700	1.20234	1.17863	1.29156	1.33174	0.11608	1.25936	1.27756	0.98204	1.38205	1.14749
764	AA	0.79844	0.76627	0.71854	0.99446	1.05136	0.96090	0.87513	0.93150	1.09181	0.89464	1.04522	0.86949	0.62538
768	AA	0.88785	0.92666	0.81800	1.29947	0.92415	0.92445	0.84323	3.57363	0.96045	1.28403	0.93747	0.90348	0.61188
834	AA	1.24740	1.07991	1.19461	1.05274	0.85773	0.91754	1.60912	0.00529	1.11503	1.05764	0.98835	0.91797	1.75361
862	AA	1.02359	1.12704	1.25780	1.08639	1.25088	1.20341	1.05157	4.20311	1.07972	0.99036	0.99224	1.16351	1.01908
873	AA	1.06043	1.53965	1.20110	0.89168	1.09647	1.04830	1.06248	0.69440	1.03169	1.05564	0.98519	1.21667	1.27607
976	AA	1.19699	0.71456	0.97332	0.64856	0.80609	0.84656	0.99592	1.79143	0.95907	0.77125	1.01519	0.83101	1.19858
602	AB	1.20141	1.25511	1.24831	1.43009	0.84849	0.83543	1.23020		1.20747	1.34290	1.02544	1.15138	0.95154
615	AB	1.30499	0.81130	1.02754	1.27322	1.15138	0.95256	1.23940	6.22897	1.09521	1.07502	1.13060	0.87643	1.35686
617	AB	1.15309	1.20236	1.35047	1.25427	1.05728	1.09252	1.50366	0.65623	1.15879	1.12477	1.02129	1.65805	1.43619
635	AB	0.90281	0.83047	0.73456	0.76492	0.83240	0.80893	0.90021	2.17402	0.97876	0.86237	1.01436	0.83700	0.95774
721	AB	1.22254	0.64206	1.03541	1.15654	1.01279	0.99601	1.22484	12.58311	1.08601	0.81074	1.11129	0.95669	1.39093
732	AB	1.05463	1.10588	1.25203	0.95267	1.56382	1.51834	0.98530	5.38979	1.07023	0.74420	0.98724	1.27271	1.30152
734	AB	1.10121	2.08792	1.69102	1.35704	1.44249	1.61265	1.08258	0.38468	1.15257	1.21369	0.90680	1.71817	1.42479
738	AB	0.82234	0.60040	0.63619	1.00051	0.79643	0.80256	0.78544	4.56027	0.73125	0.77072	1.02552	0.74795	0.83630
759	AB	1.28974	1.36734	1.41909	1.54148	1.10741	1.30550	1.37305	7.51980	1.31740	1.30677	1.12237	1.28858	1.78692
770	AB	0.87908	0.96302	0.87956	0.55492	1.52438	1.44741	0.79631	2.10467	0.89974	0.70752	0.91109	0.97906	0.84900
772	AB	0.99818	0.67656	1.10472	1.17688	0.95206	0.88041	1.11243	0.05582	1.03804	1.07411	1.13039	0.86337	1.35761
871	AB	1.16426	1.87874	1.50914	1.21614	1.24796	1.29606	1.76116	0.08980	1.37973	1.58784	1.05219	1.54378	1.94207
605	BA	0.82689	0.64199	0.56395	2.08484	0.61045	0.50393	0.84146	2.80618	0.93245	1.36010	1.06686	0.59925	0.66574
623	BA	1.29540	1.86821	1.56661	1.32418	1.12203	1.20116	1.46821	0.24401	1.18716	1.22032	1.00392	1.41190	1.51517
627	BA	1.00254	1.12004	0.94267	1.20661	1.01520	1.07550	1.00453	0.19602	0.97082	1.18662	0.86489	0.91414	1.07719
728	BA	0.83070	0.95366	0.82574	0.73691	0.92130	0.82650	0.71449	0.45356	0.91395	0.68037	0.93626	0.86345	0.70653
755	BA	1.27969	0.91764	1.06732	1.13559	1.27081	1.14573	0.98834	7.68140	0.98895	0.81754	1.11147	0.88706	1.41972
766	BA	0.93584	0.54340	0.50983	0.81194	0.65515	0.52409	0.70617	1.56085	0.79346	0.64997	0.89106	0.48049	1.11081
842	BA	1.09485	1.37707	1.49320	0.90625	0.93894	0.96577	1.27089	0.44513	1.11290	1.14122	0.95370	1.50769	1.43369
847	BA	1.03532	0.69558	0.58253	1.05746	0.81122	0.77169	0.72410	49.10654	0.65934	0.73308	0.98977	0.63847	0.48899
852	BA	1.08631	1.51575	1.26079	0.83093	1.17402	1.34565	1.08887	6.28480	1.05585	1.17446	1.00200	1.24060	1.33784
865	BA	0.87504	1.62109	1.06220	0.78270	1.16158	1.23291	0.86284	23.55121	1.06247	0.89290	0.93263	1.07504	0.98503
887	BA	0.33379	0.37011	0.58677	0.53814	0.43269	0.35929	0.24925	4.49216	0.39186	0.51064	1.15111	0.52034	0.10138
947	BA	1.22301	1.68205	1.37378	1.11821	1.24139	1.42039	1.41165	0.01904	1.08229	1.05064	0.96375	1.33888	1.29820
957	BA													
610	BB	0.83251	0.52120	0.58777	0.93357	0.77263	0.66561	0.82019	0.09210	0.95253	1.02658	1.17775	0.64887	0.58477
626	BB	1.15600	1.16293	1.35560	0.94252	1.23414	1.19077	1.34726	1.40349	1.17411	0.84081	0.99860	1.18269	1.43649
719	BB	0.69193	0.37634	0.35365	0.98880	0.59570	0.49727	0.43444	12.62842	0.65534	0.70773	1.01722	0.44948	0.24966
751	BB	1.17309	1.55976	1.30526	0.85651	1.03149	1.23442	1.31213	0.12652	1.20789	1.46028	1.01860	1.36534	1.94147
757	BB	1.06583	0.77200	0.92914	1.67442	0.72063	0.82995	0.82811	0.03213	1.09108	1.17176	0.89859	0.96468	0.96731
836	BB													
846	BB	0.67927	0.29569	0.44923	0.67586	0.67724	0.55047	0.49818	0.00413	0.66141	0.83394	1.14433	0.52682	0.36742
850	BB	1.30123	1.33194	1.30744	1.30413	0.99968	1.05799	1.38458	1.04232	1.20363	1.66961	1.01254	1.23763	1.43711
866	BB	1.21390	1.93615	1.43455	1.04766	1.44581	1.57637	1.32430	0.24071	1.14400	1.22889	1.02115	1.58670	1.25653
867	BB	1.34360	1.12034	1.45483	1.15142	1.13605	1.29712	1.23466	57.37250	1.09253	0.83896	0.87642	1.35573	1.77337
885	BB	0.98094	1.68182	1.41569		1.56746	1.42930	1.21684	0.10750	1.07637	1.11763	1.07355	1.41411	1.15282
958	BB	1.21225	0.97924	1.31646	1.08739	1.19299	1.11339	1.15932		1.09191	1.04307	1.04099	0.98159	1.38921
854	SH	1.09329	0.75244	0.94806	1.05355	0.76771	0.75937	1.40105	0.06260	1.12329	1.35948	1.10607	0.96169	1.43641
875	SH	1.17921	1.71722	1.52439	1.33784	1.04245	1.28925	1.64020	9.89044	1.28818	1.53268	0.82811	1.51484	1.85229
876	SH	0.94234	1.00862	0.94303	0.95015	1.07321	1.09011	1.01509	0.42156	1.00755	0.80133	0.97290	1.08424	1.32722
879	SH	0.73327	0.43807	1.24063	0.77977	0.80930	0.74643	0.59698	5.15497	0.83290	1.14777	0.88605	0.50281	0.63055
898	SH	0.59947	0.98468	0.59395	0.68277	0.90442	0.84631	0.48749	1.22060	0.55871	0.82970	1.18976	0.87426	0.32639
931	SH	1.16862	1.85747	1.41618	0.87817	1.45208	1.63075	1.40714	0.04358	1.29206	1.09964	0.89878	1.70159	1.55065
933	SH	1.09541	0.92870	0.98047	1.09836	0.80718	0.91528	1.16681	1.20555	1.13225	1.01043	0.95329	1.48528	1.33278
937	SH	1.15841	1.23625	1.30003	0.76601	1.08151	1.30115	1.01564	10.14945	1.09667	0.95360	0.96668	1.50142	1.21427
939	SH	1.00989	0.78643	1.02594	1.05264	0.99101	0.90779	1.10374	0.37154	1.06334	1.00054	0.97278	0.89751	1.06752
940	SH	0.90458	0.66798	0.75145	1.05676	0.79083	0.68108	1.32081	2.15077	0.98048	1.33605	1.18575	0.68277	1.09381
969	SH	1.20601	1.28378	1.20636	1.08133	1.00516	1.11843	1.27693	28.06596	1.12884	1.18094	0.95026	1.22033	1.34072
970	SH	1.05595	1.67721	1.37922	0.98733	1.68982	1.65080	1.11605	4.91675	1.05385	1.15605	1.11220	1.43092	1.10479
971	SH	1.15669	1.47928	1.26225	0.88583	1.31840	1.50843	1.15956	1.85071	1.07241	1.11406	1.00531	1.28164	0.80851

Supplementary Data S4. Raw data resulting from DNA methylation analyses within the *Avpr1a* gene in the hippocampus. Data are separately presented for each CpG site that was analysed (CpG site 1 – 16).

		Relative DNA methylation within the *Avpr1a* gene							
Animal ID	Life history	CpG site 1	CpG site 2	CpG site 3	CpG site 4	CpG site 5	CpG site 6	CpG site 7	CpG site 8
597	AA	3.81	4.15	4.41	4.97	5.35	7.58	2.32	2.09
603	AA	4.93	5.19	5.82	6.65	6.36	8.58	2.41	2.44
619	AA	4.07	5.55	6.17	7.23	7.07	9.47	2.11	2.51
722	AA	3.76	8.11	6.83	7.88	6.62	10.46	2.04	1.32
736	AA	3.26	4.53	4.85	5.52	5.54	8.27	2.05	2.14
743	AA	6.06	3.89	5.60	6.08	6.25	8.96	2.43	1.84
744	AA	5.84	4.21	4.47	3.13	5.76	8.08	2.34	2.28
764	AA	5.65	5.93	6.10	7.50	7.66	9.16	2.25	2.03
768	AA	4.07	4.86	5.08	5.95	6.24	8.44	2.19	2.18
834	AA	3.78	5.37	5.32	6.02	5.65	8.37	1.95	2.45
862	AA	3.67	4.97	5.33	5.94	6.21	8.78	2.29	1.75
873	AA	4.41	4.16	5.50	6.13	6.11	9.06	2.43	1.75
976	AA	3.78	5.06	5.81	6.25	6.24	8.37	2.36	1.85
602	AB	5.33	4.71	4.47	4.91	5.11	7.66	2.38	1.84
615	AB	4.98	3.60	3.70	5.25	5.37	8.06	1.89	3.00
617	AB	4.78	3.45	4.37	5.30	6.24	7.79	2.32	2.56
635	AB	4.95	4.23	4.30	5.97	5.75	8.76	2.23	1.99
721	AB	4.39	4.28	4.69	4.99	5.44	7.58	2.19	2.57
732	AB	3.04	4.68	5.28	5.70	5.61	7.67	2.50	2.05
734	AB	4.08						2.15	1.96
738	AB	4.65	6.85	6.79	7.49	7.40	8.74	2.63	2.02
759	AB	4.00	3.80	4.52	5.29	5.42	7.98	1.89	2.51
770	AB	4.99						2.28	2.25
772	AB	4.09						1.80	2.54
871	AB	5.20						2.45	2.73
605	BA								
623	BA	3.45	5.13	5.89	7.22	7.09	9.20	2.35	2.10
627	BA	4.84	4.49	5.25	5.31	5.90	8.67	1.91	1.75
728	BA	3.80	4.91	4.86	5.53	6.04	9.26	3.80	2.99
755	BA	3.95	3.57	4.11	4.98	5.17	7.61	1.69	2.66
766	BA	3.22	6.22	6.62	7.35	7.11	9.47	2.18	1.83
842	BA	4.69	6.24	6.40	6.82	5.53	8.84	2.31	1.80
847	BA	4.25	5.20	6.06	6.51	6.10	8.66	2.79	1.72
852	BA	4.09	3.22	4.08	4.96	5.16	7.40	2.46	2.20
865	BA	5.07	5.35	5.65	6.70	6.49	8.31	2.02	1.97
887	BA	3.38	4.15	4.51	5.35	5.31	7.51	2.36	1.62
947	BA	4.94	4.59	5.58	6.27	6.03	9.06	2.18	2.05
957	BA								
610	BB	4.55	4.49	4.16	5.43	5.34	7.40	2.70	3.06
626	BB	4.80	5.46	5.40	7.24	6.60	8.97	2.32	2.05
719	BB	3.85	4.52	4.84	5.29	5.17	7.92	2.02	1.91
751	BB	4.66	4.26	5.03	5.86	6.15	7.78	1.96	1.44
757	BB	4.55	6.14	6.63	7.22	7.17	9.40	2.42	2.23
836	BB								
846	BB	3.97	4.02	4.33	5.54	5.39	7.13	2.03	2.53
850	BB	4.90	5.34	6.49	6.50	6.30	8.67	1.98	2.35
866	BB	3.86	4.17	4.29	5.39	5.17	8.14	2.51	2.06
867	BB	3.79	3.81	4.59	6.57	5.54	8.81	2.10	2.39
885	BB	5.21	2.88	5.37	5.09	5.99	8.97	2.05	2.66
958	BB	4.84	5.04	5.33	6.00	5.92	8.37	2.08	1.91
854	SH	5.26	3.66	4.06	5.27	5.02	7.50	2.06	2.16
875	SH	4.22	6.02	6.27	7.55	7.55	9.98	2.58	2.23
876	SH	4.54	4.09	6.22	5.58	5.19	8.53	2.02	2.07
879	SH	4.18	4.12	4.66	5.02	5.25	8.26	1.96	2.34
898	SH	4.59	4.44	4.03	5.43	5.22	7.60	1.91	2.23
931	SH	3.96	6.38	7.01	7.88	7.45	9.31	1.85	2.45
933	SH	4.54	6.33	6.62	7.09	7.15	8.99	2.31	2.63
937	SH	4.53	4.33	5.23	4.97	5.57	8.55	1.88	1.74
939	SH	4.75						2.28	1.94
940	SH	5.65	4.25	4.88	5.88	6.14	8.47	2.89	2.32
969	SH	5.40	6.50	6.90	7.57	7.12	9.46	2.69	2.96
970	SH	4.45	6.34	6.74	7.74	6.79	9.16	2.09	2.16
971	SH	4.07	4.53	4.30	5.60	5.78	8.16	2.15	2.44

Supplementary Data S4 (continued).

Animal ID	Life history	Relative DNA methylation within the *Avpr1a* gene							
		CpG site 9	CpG site 10	CpG site 11	CpG site 12	CpG site 13	CpG site 14	CpG site 15	CpG site 16
597	AA	0.57	1.43	2.07	1.26	1.26	1.12	1.10	1.25
603	AA	0.70	1.23	1.86	0.78	1.22	0.95	1.37	1.09
619	AA	0.80	1.68	2.32	0.84	1.10	0.77	1.92	1.60
722	AA	1.08	2.29	2.38	0.00	0.83	0.99	1.29	2.50
736	AA	1.18	1.38	1.42	0.64	1.23	0.00	1.24	0.91
743	AA	0.89	1.81	2.18	0.51	0.63	0.85	1.91	1.37
744	AA	0.88	1.86	2.98	0.60	1.25	0.76	1.13	1.47
764	AA	0.77	1.52	2.23	0.87	0.68	0.50	1.43	1.36
768	AA	0.46	1.66	1.90	0.70	1.16	0.54	1.32	1.48
834	AA	0.86	2.12	1.89	0.79	0.96	0.00	1.02	1.64
862	AA	0.80	1.39	2.43	1.21	1.17	0.63	1.28	2.02
873	AA		1.08	2.06	1.37	0.84	0.00	1.49	1.48
976	AA	0.67	1.43	2.00	0.00	0.85	1.16	1.53	1.62
602	AB	0.66	1.75	2.18	1.26	0.95	1.00		0.00
615	AB	1.16	1.72	2.23	0.84	1.06	1.01	1.85	1.61
617	AB	0.70	2.39	3.03	0.89	0.00	0.00	1.09	1.30
635	AB	0.89	1.67	1.91	0.55	0.90	0.64	1.29	1.64
721	AB	0.71	1.94	1.71	1.11	1.34	0.95	1.02	1.76
732	AB	0.89	1.25	1.56	0.58	1.18	0.72	1.59	1.41
734	AB	0.75	2.06	1.90	0.97	0.99	0.60	1.31	1.61
738	AB	0.60	1.21	1.82	0.00	0.85	0.68	1.50	1.74
759	AB	0.48	1.56	2.09	0.67	0.73	0.80	1.05	1.34
770	AB	0.79	1.90	2.58	0.80	0.79	0.00	1.30	1.61
772	AB	0.60	1.68	2.03	0.90	0.80	0.80	1.38	1.18
871	AB	0.76	1.09	1.97	0.73	0.85	0.80	1.60	1.35
605	BA								
623	BA	0.00	1.77	1.66	1.34	2.12	1.24	1.67	1.26
627	BA	0.67	1.66	2.08	0.99	1.13	0.00	0.00	1.59
728	BA	1.73	2.63	2.25	1.64	2.55	1.69	3.45	2.67
755	BA	0.74	1.25	2.50	0.74	0.84	0.84	1.08	1.67
766	BA	0.85	0.96	1.80	0.74	1.16	0.69	1.39	1.38
842	BA	0.76	1.76	2.08	1.03	1.23	0.94	0.78	1.26
847	BA	0.86	1.35	1.97	0.87	1.63	0.67	1.50	1.38
852	BA	1.01	1.47	1.67	0.53	0.80	0.00	0.69	1.21
865	BA	0.69	1.06	1.57	0.72	1.05	0.67	0.00	1.50
887	BA	0.92	1.12	2.00	0.96	1.23	0.78	1.14	1.33
947	BA	0.72	1.23	1.79	1.12	0.83	1.14	1.39	1.71
957	BA								
610	BB	0.97	1.48	2.19	0.66	1.10	1.31	1.60	1.06
626	BB	0.85	2.18	1.91	1.31	0.98	0.60	1.59	1.72
719	BB	0.65	1.34	1.98	0.79	0.90	0.59	1.44	0.77
751	BB	0.83	0.94	2.52	0.99	0.89	0.00	1.42	1.20
757	BB	0.88	1.56	1.31	0.00	0.00	0.00	0.00	1.10
836	BB								
846	BB	0.77	1.94	2.21	0.85	0.71	0.74	1.30	1.63
850	BB	0.81	1.47	1.38	0.91	0.71	0.99	1.36	1.50
866	BB	0.72	1.40	2.01	0.70	1.18	1.02	1.09	1.54
867	BB	0.78	1.37	1.97	0.00	0.78	0.97	0.00	1.26
885	BB	0.70	1.62	1.73	1.11	1.09	0.00	1.20	1.06
958	BB	0.94	1.75	2.71	0.70	1.43	1.04	1.21	1.58
854	SH	0.58	1.36	2.33	1.52	1.10	0.51	1.66	1.26
875	SH	1.14	0.00	2.16	0.76	1.05	0.90	1.49	1.13
876	SH	0.81	1.66	1.89	0.85	0.97	0.00	1.49	1.34
879	SH	0.61	1.61	2.59	0.77	0.71	0.00	1.05	0.94
898	SH	0.51	1.23	1.91	0.72	1.11	1.00	1.31	1.34
931	SH	1.53	1.81	2.67	0.90	1.41	1.14	1.61	1.18
933	SH	1.10	1.43	2.44	0.93	1.36	1.80	1.34	1.70
937	SH	0.67	1.34	1.95	0.57	0.79	0.71	0.80	1.60
939	SH	0.75	1.20	1.52	0.54	0.71	0.76	1.25	1.47
940	SH	1.15	2.04	2.74	1.29	1.67	0.92	1.48	1.54
969	SH	0.83	1.42	2.29	1.08	1.36	1.25	1.83	1.81
970	SH	1.10	1.28	1.81	0.71	0.90	0.54	1.30	1.46
971	SH	0.81	1.33	2.23	0.44	1.53	0.72	1.48	1.31
